# Mutational landscape of SARS-CoV-2 genome in Turkey and impact of mutations on spike protein structure

**DOI:** 10.1371/journal.pone.0260438

**Published:** 2021-12-06

**Authors:** Ozden Hatirnaz Ng, Sezer Akyoney, Ilayda Sahin, Huseyin Okan Soykam, Gunseli Bayram Akcapinar, Ozkan Ozdemir, Derya Dilek Kancagi, Gozde Sir Karakus, Bulut Yurtsever, Ayse Sesin Kocagoz, Ercument Ovali, Ugur Ozbek

**Affiliations:** 1 Department of Medical Biology, Acibadem Mehmet Ali Aydinlar University School of Medicine, Istanbul, Turkey; 2 Acibadem Mehmet Ali Aydinlar University Rare Diseases and Orphan Drugs Application and Research Center (ACURARE), Istanbul, Turkey; 3 Department of Biostatistics and Bioinformatics, Institute of Health Sciences, Acibadem Mehmet Ali Aydinlar University, Istanbul, Turkey; 4 Department of Medical Biotechnology, Institute of Health Sciences, Acibadem Mehmet Ali Aydinlar University, Istanbul, Turkey; 5 Department of Medical Genetics, Acibadem Mehmet Ali Aydinlar University School of Medicine, Istanbul, Turkey; 6 Department of Genome Studies, Institute of Health Sciences, Acibadem Mehmet Ali Aydinlar University, Istanbul, Turkey; 7 Acibadem Labcell Cellular Therapy Laboratory, Istanbul, Turkey; 8 Department of Infectious Diseases and Clinical Microbiology, Acibadem Mehmet Ali Aydinlar University School of Medicine, Istanbul, Turkey; University of Hail, SAUDI ARABIA

## Abstract

The Coronavirus Disease 2019 (COVID-19) was declared a pandemic in March 2020 by the World Health Organization (WHO). As of May 25th, 2021 there were 2.059.941 SARS-COV2 genome sequences that have been submitted to the GISAID database, with numerous variations. Here, we aim to analyze the SARS-CoV-2 genome data submitted to the GISAID database from Turkey and to determine the variant and clade distributions by the end of May 2021, in accordance with their appearance timeline. We compared these findings to USA, Europe, and Asia data as well. We have also evaluated the effects of spike protein variations, detected in a group of genome sequences of 13 patients who applied to our clinic, by using 3D modeling algorithms. For this purpose, we analyzed 4607 SARS-CoV-2 genome sequences submitted by different lab centers from Turkey to the GISAID database between March 2020 and May 2021. Described mutations were also introduced *in silico* to the spike protein structure to analyze their isolated impacts on the protein structure. The most abundant clade was GR followed by G, GH, and GRY and we did not detect any V clade. The most common variant was B.1, followed by B.1.1, and the UK variant, B.1.1.7. Our results clearly show a concordance between the variant distributions, the number of cases, and the timelines of different variant accumulations in Turkey. The 3D simulations indicate an increase in the surface hydrophilicity of the reference spike protein and the detected mutations. There was less surface hydrophilicity increase in the Asp614Gly mutation, which exhibits a more compact conformation around the ACE-2 receptor binding domain region, rendering the structure in a “down” conformation. Our genomic findings can help to model vaccination programs and protein modeling may lead to different approaches for COVID-19 treatment strategies.

## Introduction

The Coronavirus Disease 2019 (COVID-19) is caused by severe acute respiratory syndrome coronavirus 2 (SARS-CoV-2) and has been declared a pandemic by the World Health Organization (WHO, 2020). Until September 2021, more than 229.3 million people have been affected and there are over 4.7 million confirmed deaths around the world [1]. SARS-CoV-2 genome is a single-stranded, positive-sense RNA, around 30-kilo bases (kb) long, and encoding 9860 amino acids [2]. The SARS-CoV-2 genomic RNA consists of 14 Open Reading Frames (ORFs). Of the main ORFs, ORF1a and ORF1b cover almost ⅔ of the viral genome and encode polyproteins pp1a and pp1b that produce non-structural proteins (NSPs). These NSPs are responsible for the replication of the viral genome following infection. The rest of the ORFs are coding the four structural proteins: envelope (E), spike (S), membrane (M), and nucleocapsid (N). S, M, and E form the envelope of the virus; S and M are also transmembrane proteins involved in virus replication. The S protein gives the envelope a crown shape like structure, which is also the origin of the name *coronavirus*. These proteins are needed for virion formation inside the host cell and release of the virus out of the cell for new infection targets. The spike protein is the main part of the virus responsible for infection of the host cells. It is a glycoprotein consisting of two subunits (S1 and S2) [3–5]. SARS-CoV-2 infects the respiratory cells via binding to the Angiotensin-Converting Enzyme 2 (ACE2) receptor [6]. S1 recognizes the ACE2, and S2 plays a role in the fusion to the host cell membrane. Due to its vital role, the Spike protein is one of the most studied regions of the virus. Many studies are focused on the structural aspects of the Spike-ACE2 interaction and the effects of mutations on spike structure, confirmation, and function (i.e., antigenicity) in order to understand the mechanistic aspects of this interaction, evaluate the effect of neutralizing antibodies, vaccinate candidates, and project the possible impacts of virus evolution. Therefore, with the novel mutations, it is of crucial importance to determine and understand the potential impact of these on Spike structure and function [7–12]. SARS-CoV-2 is closely related to severe acute respiratory syndrome (SARS) and Middle East Respiratory Syndrome (MERS)-CoV. Due to the nature of viruses, random genomic variations are inevitable. Exploring these variations can reveal the spread map of a virus [13]. Viral genome sequencing studies of the SARS-CoV-2 virus are ongoing across the world and many different strains have been described with respect to the reference genome. One of the first variations that spread vigorously across countries was Asp614Gly at the spike protein, with this mutation showing higher viral loads than the reference virus from Wuhan, China [14].

Worldwide viral genome data is submitted to the Global Initiative on Sharing All Influenza Data (GISAID) database [15]. As of 24 May 2021, the GISAID database has created eight major groups of lineages for SARS-CoV-2; G, GH, GR, GRY, GV, L, S, and V. The variant lineages that do not fall into any of these clades are classified as other (O). Recently, the GK clade, which is also known as the delta variant, has also been added as the ninth GISAID clade [15]. According to their aggressive spread and increased death rates, there are four different variances of SARS-CoV-2 identified as “variants of concern, (alpha, beta, gamma, and delta)”. Additionally, “Variants of Interest” (VOI) are also specified in the late 2020 and currently, there are two main VOI (Lambda and Mu) that show a tendency to spread but their effects on transmissibility, disease severity, immune or therapeutic escape are not fully understood. These variants are diverse from each other due to the mutations which occur in spike glycoprotein [16–18]. Both VOC and VOI are named after where they were first recognized [19]. Recently, the WHO also updated the nomenclature of these country-specific names [20]. Today’s most abundant variant and one of the variants of concern is known to originate from the UK, namely B.1.1.7 (Alpha). Another variant of concern, B.1.351 (Beta), originates from South Africa. Studies showed vaccines may be less effective against these mutant forms of the SARS-CoV-2 [21, 22]. According to the PANGO lineage database [23], the B.1.9.5 European sublineage which was detected firstly in March 2020 is highly represented in Turkey with a rate of 27.0%. As of today, any other variant originating from Turkey has not been described.

Previously, SARS-CoV-2 genome analyses were performed with a limited number of sequences in Turkey [24–27]. The aim of this study was to analyze the distribution of the VOC, the clades, and the most abundant variations in 4607 sequences submitted to the GISAID database from Turkey (accession date: May 25th, 2021). Moreover, these results were compared with data of geographically separated regions in the World such as the United States of America, Europe, and Asia. Additionally, the SARS-CoV-2 virus genomes obtained from 13 Turkish patients from our clinics were analyzed to determine the variations and to explore the impact of these mutations on SARS-CoV-2 spike protein structure through *in silico* methods using molecular modeling and molecular dynamics.

## Materials and methods

### Viral genome data and patient samples

We tabulated all SARS-CoV-2 genome data submitted from Turkey to the GISAID database [15] between March 16^th^, 2020, and May 25^th^, 2021 (n = 4607). The highest submitter was the Turkish Ministry of Health (n = 4168), which received samples from the whole country. The collection dates were divided into three-month periods as follows: March-May 2020 n = 327 sequences; June-August 2020 n = 83 sequences; September-November 2020 n = 58 sequences; December 2020-February 2021 n = 2549 sequences; and March-May 2021 n = 1589 sequences. Thirteen of these samples were sequenced by our group at the Medical Genetics Department of Acibadem University, Istanbul, Turkey. The nasopharyngeal and oropharyngeal cavity samples were obtained from 13 patients (eight males and five females, sequence IDs are provided in S1 Table) diagnosed as COVID-19 positive by real-time PCR between April-May 2020. All patients were hospitalized in an Acibadem University affiliated hospital and received COVID-19 treatment according to the Turkish Ministry of Health’s set of recommendation guidelines, which were developed by the national Coronavirus Scientific Advisory Board based on the global data. The Acibadem University Medical Research Ethical Committee approved this study (ATADEK-2020/05/41) and all patients signed informed consent forms. The most common admittance symptoms among the patients were fever (75%) and coughing (75%). Patients also had difficulty in breathing and felt fatigued. Six of the patients had a known history of previous chronic diseases (Table 1).

**Table 1:** Clinical features of patients included in this study.

Sample ID	Sex	Age	First symptom	Hospital Admission	Pre-clinical Findings	PCR result	CT image	Chronic Disease History	Drug Treatment	Current Status of the Patient	Contact/ Travel History
**ACUTG-1**	M	50	Unknown	13.04.2020	Fever, cough	Positive	Typical ground glass appearance	No known chronic disease	Hydroxychloroquine, Azithromycin	Discharged from hospital	Unknown
**ACUTG-2**	M	57	03.04.2020	06.04.2020	Fever, cough	Positive	Indeterminate appearance	Vasomotor and allergic rhinitis	Tocilizumab, Favipiravir, Hydroxychloroquine	Discharged from hospital	Unknown
**ACUTG-3**	M	42	Unknown	07.04.2020	Fever, cough	Positive	Indeterminate appearance	No known chronic disease	Hydroxychloroquine, Azithromycin, Oseltamivir	Discharged from hospital	Contact
**ACUTG-4**	M	44	24.03.2020	03.04.2020	Cough, difficulty in breathing, cold, shivering, muscle pain	Positive	Typical ground glass appearance	No known chronic disease	Azithromycin, Oseltamivir, Hydroxychloroquine	Discharged from hospital	Unknown
**ACUTG-5**	M	34	21.04.2020	22.04.2020	High fever, cough	Positive	Negative for pneumonia	Diabetes mellitus	Azithromycin, Oseltamivir, Hydroxychloroquine	Discharged from hospital	Unknown
**ACUTG-6**	M	66	Unknown	18.04.2020	Chest burning, fever, cough	Positive	Typical ground glass appearance	Diabetes mellitus	Hydroxychloroquine, Favipiravir	Discharged from hospital	Unknown
**ACUTG-7**	F	28	03.05.2020	05.05.2020	Sore throat, myalgia, diarrhea	Positive	NA	Unknown	Hydroxychloroquine	Discharged from hospital	Contact
**ACUTG-8**	F	37	09.04.2020	11.04.2020	Cough, shortness of breath, fatigue	Positive	Negative for pneumonia	Irritable bowel syndrome	Hydroxychloroquine	Discharged from hospital	Unknown
**ACUTG-9**	M	50	Unknown	05.05.2020	NA	Positive	Typical ground glass appearance	No known chronic disease	NA	Discharged from hospital	Unknown
**ACUTG-10**	M	56	Unknown	19.04.2020	Fever, malaise, and fatigue	Positive	Typical ground glass appearance	Hypertension	Azithromycin, Hydroxychloroquine	Discharged from hospital	Unknown
**ACUTG-11**	F	23	13.04.2020	15.04.2020	Fever, fatigue, cough	Positive	Negative for pneumonia	No known chronic disease	NA	Discharged from hospital	Unknown
**ACUTG-12**	F	23	Unknown	06.05.2020	Fever, cough, fatigue	Positive	Typical ground glass appearance	No known chronic disease	Hydroxychloroquine	Discharged from hospital	Unknown
**ACUTG-13**	F	69	Unknown	20.04.2020	High fever, chest pain, difficulty in breathing	Positive	Typical ground glass appearance	Hypertension, diabetes, embolism	Favipiravir, Hydroxychloroquine, Azithromycin, Tocilizumab	Discharged from hospital	Unknown

The CT images are grouped according to the recommendations of the Radiological Society of North America Expert Consensus Statement^39^. F: female, M: male, CT: computed tomography, NA: no data available.

### Cell culture and RNA extraction

Collected samples were immediately transferred to an Acıbadem LabCell Cellular Therapy Laboratory BSL-III unit in a transfer medium and cultured on the Vero cell line as described previously [28]. The viral RNA extraction was performed with the Quick-RNA Viral Kit (Zymo Research, USA) and RNA concentration was determined by using the NanoDrop 2000 (ThermoFisher Scientific, USA). The samples were stored at -80°C until further studied.

### Viral genome sequencing

Following viral RNA extraction, genome sequencing and data analysis were performed consecutively. Library preparation was performed via CleanPlex SARS-CoV-2 Research and Surveillance NGS Panel (Paragon Genomics, USA) according to the manufacturer’s protocol. A multiplex PCR reaction with a 2-pool design was chosen to obtain the full coverage of the SARS-CoV-2 genome. Library construction was performed with CleanPlex^®^ Dual-Indexed PCR Primers for Illumina^®^ (Paragon Genomics, USA) by combining the i5 and i7 primers. AgencourtTM AMPureTM XP (A63880) beads (Beckman Coulter Inc.) were used for the purification steps. The purification process was performed with a DynaMag-96 side magnet (ThermoFisher Scientific, USA). T100 Thermo Cycler (Bio-Rad Laboratories, Inc) was used for the PCR and incubation steps. The sequencing was performed with an Illumina MiSeq instrument with paired-end 131 bp long fragments by a service provider.

### Data analysis

#### Mutation analysis

Viral genome sequencing data processing was performed with the pipeline summarized in S1 Fig. Since the default option for the *Homo sapiens* genome is diploid, the “sample-ploidy” option was applied as “1” at the variant calling step of the virus analysis. The complete genome of severe acute respiratory syndrome coronavirus 2 isolate Wuhan-Hu-1 (NC_045512.2) was used as a reference [2]. Detected mutations were confirmed with Integrative Genomics Viewer (IGV) [29]. In addition, when the mutation coincided with the upstream or downstream of the fragment or reading depth was low, this mutation was eliminated. To show the clear diversity of the variation, the clade system introduced by the GISAID was used as described previously [30]. We checked a total of 79,884 viral genome sequences uploaded to the GISAID database [15] as of August 2020 to determine if the mutations that were detected in the viral genome sequences obtained from 13 patients were previously detected or not.

#### Multiple alignment and construction of the phylogenetic trees

A multi-FASTA file consisting of 4607 viral genomes from Turkey was downloaded from GISAID [15]. Viral genome sequences were aligned with the NC_045512.2 reference genome [2] and to each other via multiple sequence alignment performed using MAFFT v7 [31]. The maximum likelihood phylogenetic tree was constructed with 1000 ultrafast bootstraps via IQTREE software [32]. A total of 286 DNA models were tested for a proper model of substitution via the ModelFinder function of IQTREE. As a best-fit model according to Bayesian information criterion scores, the GTR+F+R10 model was used for tree construction. Tree visualization was made with the iTOL tool [33].

#### Structural analysis of the SARS-CoV-2 spike glycoprotein mutations

The spike glycoprotein structure in a closed state in the COVID-19 archive (PDB ID: 6VXX) [34] prepared by CHARMM-GUI [35, 36] was used as the starting structure for the introduction of mutations. The 6VXX Cryo-EM structure of the spike glycoprotein deposited in the PDB database contains missing residues. Structure files obtained from CHARMM-GUI based on the 6VXX reflect the spike protein’s glycosylated form with the addition of missing residues. Single mutations Ala222Val, Tyr265Cys, and Asp614Gly were introduced to the spike glycoprotein structure via Visual Molecular Dynamics (VMD) software with a Mutator Plugin [37]. The ConSurf web server (http://consurf.tau.ac.il) was used to determine the conserved and variable regions on the protein structure [38].

#### Molecular Dynamics (MD) analysis of SARS-CoV-2 spike mutations

Molecular Dynamics simulations were performed on the SARS-CoV-2 spike protein in the closed state from the COVID-19 archive (http://www.charmm-gui.org/docs/archive/covid19) [35] to study the effects of the individual mutations on the closed conformation since the open conformation is the dominant form interacting with the ACE2 receptor. A fully glycosylated S protein head-only model (residue 1–1146) based on 6VXX was used as the initial structure. To reduce the computational burden, the sugars were removed since single mutations are not located on the glycosylated residues or regions in their vicinity. Single mutations Ala222Val, Tyr265Cys, and Asp614Gly, were introduced to the structure using the CHARMM-GUI Solution Builder module [36].

Throughout the MD simulation, the CHARMM36 force field [39] for proteins, lipids, and carbohydrates was used with the TIP3P water model [40]. The protein structures were embedded in a rectangular water box with an edge distance of 10 A° and neutralized with 0.15 M KCl. Periodic Boundary Conditions (PBC) [40] and Particle Mesh Ewald (PME) methods were applied [41, 42]. Long-range electrostatic interactions were calculated by PME [41, 42] with a maximum grid spacing of 1 A°. MD simulations were run at 310.15 K using the Kusing NAMD Software Package [43], starting with the input files generated in CHARMM-GUI. Systems were minimized for 10000 steps, equilibrated at NVT (constant particle number, volume, and temperature) for 250 ps with a 2fs timestep. Production runs were performed at NPT (constant particle number, pressure, and temperature) for 20 ns with a 4fs timestep. The MD simulations were extended to 20 ns to observe the stability of the changes inferred by the mutations further in a relatively long timeframe. During equilibration runs, collective variable restraints were used to slowly release the system to facilitate stable simulation. Each simulation was performed at least twice.

Root mean square deviation (RMSD) is an indicator of the large structural changes in the protein and is used to measure the scalar distance between atoms of the same type for two structures [44]. Total RMSD values of the spike protein’s alpha carbon atoms and its mutations after the equilibration were calculated using VMD analysis tools. Additionally, the RMSD of the residues between 439 to 501 were calculated. This region includes the ACE-2 receptor-binding domain. Root mean square fluctuation (RMSF) is an indicator of individual residue flexibility, or how much a popular residue moves during a simulation. Average RMSF values throughout 20 ns trajectories were calculated for each protein SASA, as described in section 2.5 at t = 0 and t = 20.

Normal Mode Analysis (NMA) is an analysis method to study the dynamic aspects of large conformational changes seen in a protein. Bio3D Enhanced NMA analysis module was used to calculate the deformation energies reflecting the amount of local flexibility; atomic fluctuations from the first three modes along the 20 ns trajectories [45, 46].

## Results

### The variant distribution of SARS-CoV-2 genomes in Turkey

**Fig 1:**
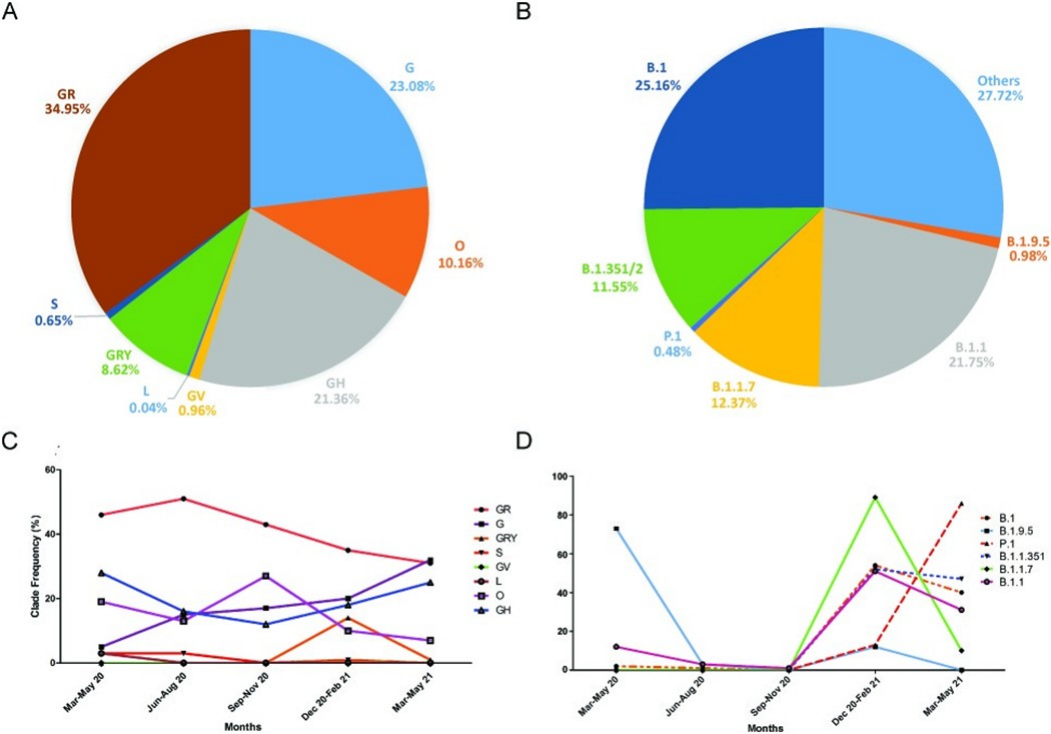
Distribution of clades and lineages among 4607 genomes. (A) Pie chart of the most common GISAID clades [15] (B) Pie chart of the most common lineages. (C) Line graph of clade frequencies comparisons with different periods of the pandemic. (D) Line graph of lineage frequencies comparison with different timelines of the pandemic. B.1.1.7; UK variant, P.1; Brazil variant, B.1.351/2; South Africa variant.

Among the 4607 viral genomes, the number of constant sites was 26376/29993 (= 88% of all sites) and parsimony-informative sites were 2270. In total, 5898 distinct site patterns were detected. The distribution of clades among the 4607 whole viral genomes (S1 Table), including 13 SARS-CoV-2 genome sequences obtained from our clinic were analyzed and seven different clades were detected (Fig 1A). The most abundant clade was GR (n = 1610), followed by G (n = 1063), GH (n = 984), GRY (n = 397), GV (n = 44), and S (n = 30). The L clade consisted of ten samples and there were no samples in the V clade. Ten percent of the samples (n = 468) did not fall into a known clade and were grouped as Others (O, Fig 1A). Among the 13 genome sequences, all except one were also classified as O, and the genome sequence of the patient coded ACUTG-5 was classified as GR. The most abundant variant was B.1, which was detected in 1159 (25.16%) genomes, followed by B.1.1 in 1002 genomes (21.75%). On the other hand, the UK variant, B.1.1.7, was detected in 570 genomes (12.37%, Fig 1B). The variant B.1.9.5, which was determined to be detected the most in Turkey, was found in 45 genomes. The Indian variant B.1.617.2 (Delta) which currently corresponds to GK clade was detected only in one genome.

We also analyzed the timeline differences in the frequencies of clades and lineages according to their appearances over 15 months (March 2020-May 2021). The GR, G, and GH clades were detected almost every month with high frequencies and the GR was the most abundant (Fig 1C). The highest peak of the GR clade was detected between June-August 2020. The G clade has been continuously increasing so that by May 2021, it was the highest clade seen in Turkey. The GH clade frequency was in a decreasing trend, but by September-November 2020 it started to increase until the end of May 2021. The GRY clade was only detected between September 2020-February 2021 and the clade was almost lost by May 2021. The S, GV, and L clades were detected only in a limited number of samples (Fig 1C). The timeline differences in the frequency of variant lineages between March 2020-May 2021 were illustrated in Fig 1D. The first lineage with the highest frequency was B.1.9.5. It showed a fast decrease by June 2020, then a slight increase in December 2020, and it was lost by May 2021. By September 2020, the frequency of all the lineages was increasing, with B.1.1.7 (Alpha) being the highest, followed by B.1, B.1.351, and B.1.1. The P.1 (Gamma) lineage was first detected in September 2020 and in December 2020 the increase accelerated. By May 2021, it was the most abundant lineage detected in Turkey. There was an obvious decrease in the B.1.1.7 lineage in December 2020-February 2021 (Fig 1D).

**Fig 2:**
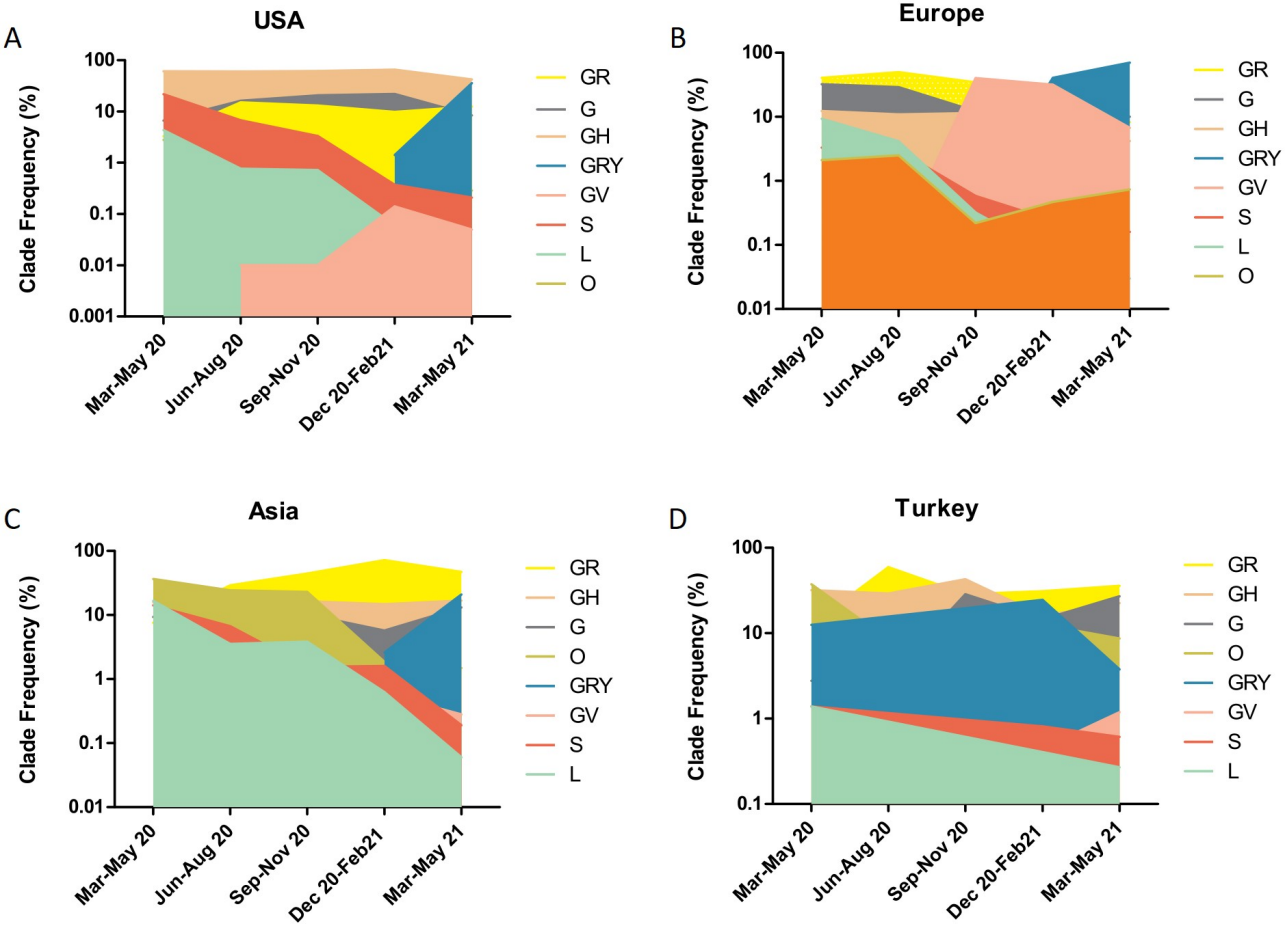
Clade distributions among different regions of the world. The distribution of seven different clades analyzed between March 2020-May 2021 divided into three months periods in A) USA, B) Europe, C) Asia, D) Turkey.

The clade distributions among the three regions were compared to the distribution in Turkey as well. As in Turkey, the highest clade was GR in Asia, whereas in the USA the highest peak was found in GV and in Europe in GRY (Fig 2).

**Fig 3:**
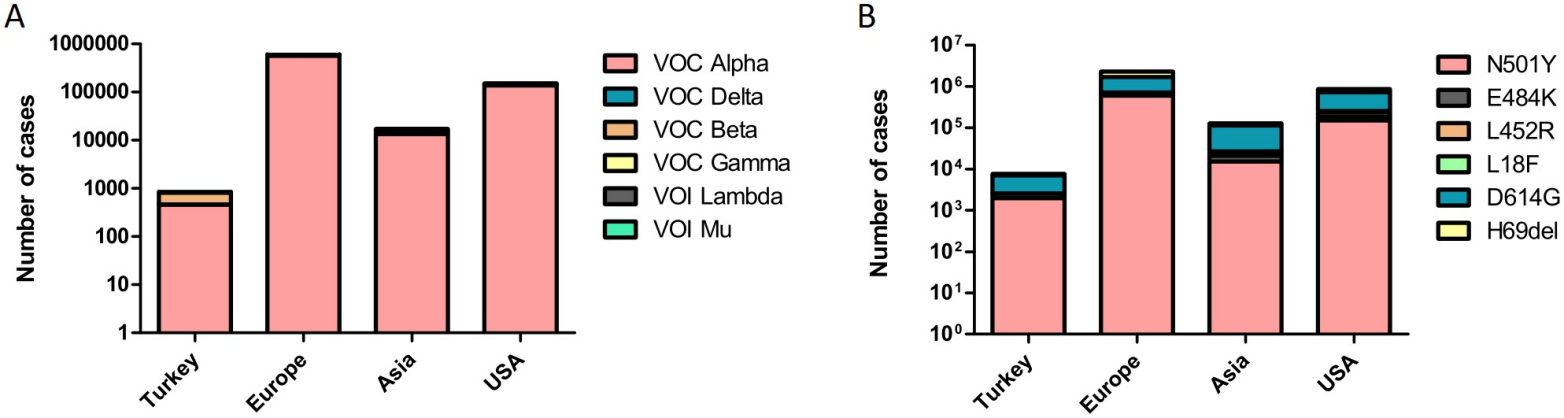
The distribution of the VOC and VOI in three different regions of the world compared to Turkey. (A) Column graph of VOCs and VOIs among Turkey, Asia, Europe, and the USA. (B) Column graph of important spike amino acid changes among Turkey, Asia, Europe, and the USA.

We also analyzed the VOC and VOI numbers in Turkey and compared them with the distribution in other regions of the World (Fig 3A). The analysis showed that all the VOC and VOI were detectable in Europe and in the USA. In Asia, only the Mu variant was absent and in Turkey, there was no VOI reported until the 25th of May, 2021. The Alpha variant was the dominant VOC in these four regions, but the Alpha and the Beta VOCs had quite close numbers of samples in Turkey. The Delta variant was only reported once in Turkey in the timeline of this study and when compared to the other three regions, Turkey had the lowest numbers for the Delta variant. The distribution of specific amino acid changes in Spike glycoprotein, which are already related with increasing in transmissibility, showed that the N501Y and D614G mutations were widely detected in Europe when compared to other regions (Fig 3B). The D614G was the most seen mutation in all four regions. All mutations except L18F were distributed well in all four regions but the number of L18F mutations was close to zero in Turkey when compared to other continents (Fig 3B).

**Fig 4:**
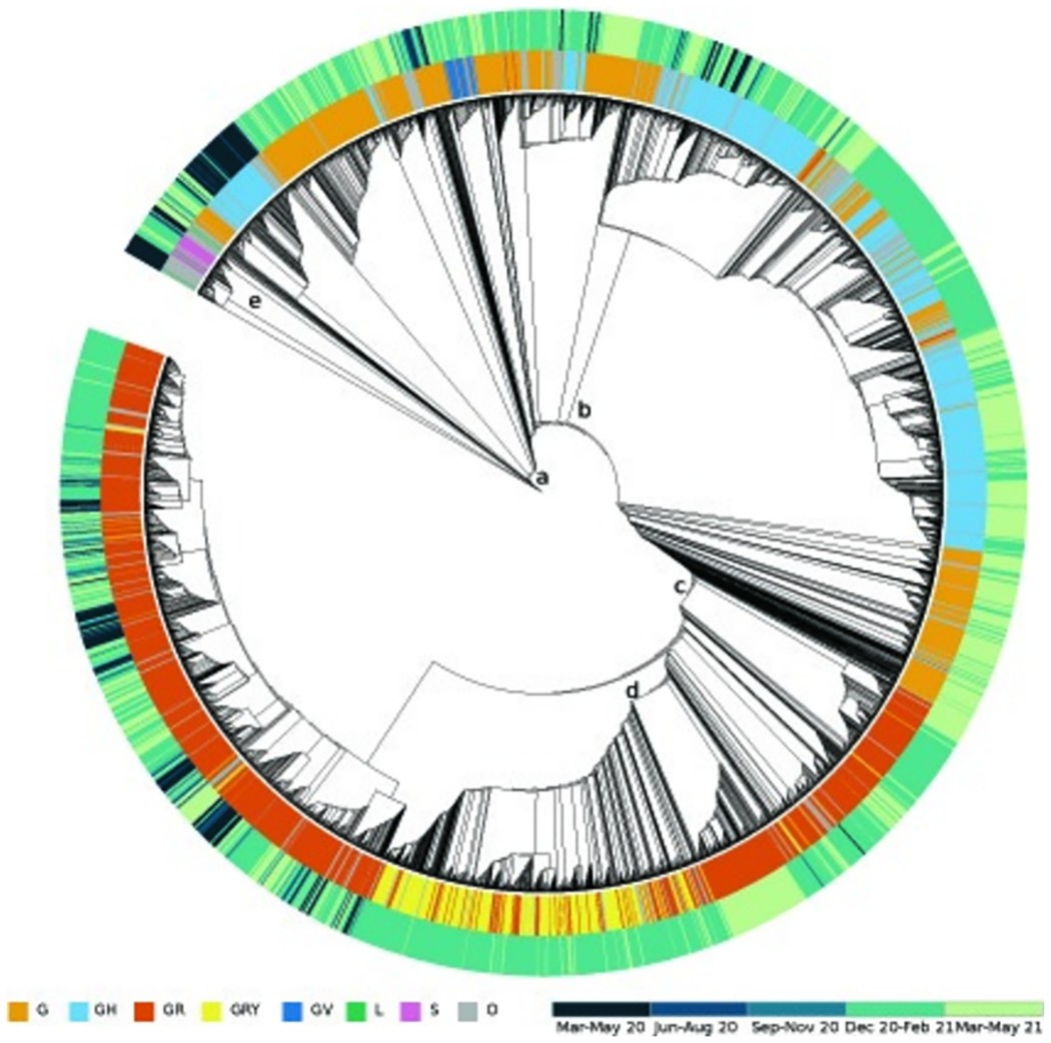
Circular representation of the phylogenetic tree of 4607 viral genomes from Turkey. The inner circle defines GISAID clades with different colors for each individual data point. The outer circle defines the timelines of the collection of data and each time period is represented by different color codes. The branches of the main clades that were discussed in the manuscript are represented by letter codes (a) Main branch of G clade, (b) GH clade, (c) GR clade, (d) GRY clade, and (e) the branch of the genome sequences of 13 patients in our center.

The phylogenetic tree analysis presents the clade distributions and collection dates (Fig 4). The phylogenetic tree diverges into three main branches regarding the lineages detected in different timelines. The seven different clades and samples other than these (O) were illustrated in the inner ring. The collection dates are represented in the outer ring of the phylogenetic tree with five different colors (Fig 4). The G clade (branch a) was detected throughout the whole pandemic in Turkey. The GH clade (branch b) was mostly detected after December 2020 and continues to be detected today. The GR (branch c) and the GRY (branch d) clade were found in high frequencies throughout the whole pandemic in Turkey, especially between December 2020-February 2021. In the phylogenetic tree, 12 out of the 13 cases sequenced in our clinic were grouped in the same branch (branch e) and located at the beginning of the pandemic (March-May 2020), whereas the genome sequence of ACUTG-5 was located in a different branch.

### Detected mutations

**Fig 5:**
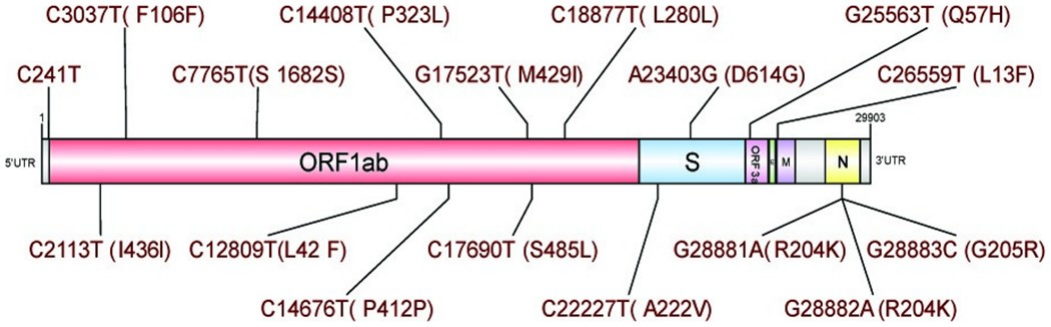
Localization of the mutations on the SARS-CoV-2 genome. The mutations were detected among the genome sequences of 13 patients who applied to our department. The figure was drawn by DOG v2 [47]. Different regions of the viral genome are represented by different colors. Open Reading Frame (ORF), envelope (E), spike (S), membrane (M), and nucleocapsid (N). Amino acid changes are shown in brackets.

In the analyses of the genome sequences obtained from 13 patients from our clinic, following a quality check and filtering, 70 single nucleotide variations (SNV) in 17 different nucleotide positions were obtained (Table 2). Eleven of these mutations were missense, five of them were synonymous, and one was in the 5’UTR. The detected mutations were mostly located on the ORF1ab gene (45%). We have also determined variations on S, M, ORF3a, and N gene regions (Fig 5). The most common mutations were C17690T (69.2%), C2113T (61.5%), C241T (53.8%) and G25563T (53.8%) and in 38.5% of the cases these mutations were detected together [26]. Three consecutive mutations of the N gene (G28881A, G28882A, and G28883C) were observed in four (ACUTG-5, ACUTG-6, ACUTG-8, and ACUTG-13) of the 13 SARS-CoV-2 genome sequences. Only one mutation (C241T) was in a non-coding region. The mutation A23403G, known as D614G, was detected in genome sequences of two of our patients (ACUTG-1 and ACUTG-5).

**Table 2:** Detailed information on detected mutations.

Nucleotide position	Gene/region	Gene product	Nucleotide exchange (Ref/Alt)	Amino acid exchange	Mutation type	Conservation among 9332 genome sequences	Frequency in this study
**241**	5’ UTR	Untranslated region	C/T	-	-	59.52%	53.8%
**2113**	ORF1ab	Nsp2	C/T	Ile436Ile	synonymous	98.07%	61.5%
**3037**	ORF1ab	Nsp3	C/T	Phe106Phe	synonymous	61.27%	30.8%
**7765**	ORF1ab	Nsp3	C/T	Ser1682Ser	synonymous	98.18%	15.4%
**12809**	ORF1ab	Nsp9	C/T	Leu42Phe	missense	98.47%	7.7%
**14408**	ORF1ab	RNA-dependent RNA Polymerase	C/T	Pro323Leu	missense	61.01%	23.1%
**14676**	ORF1ab	RNA-dependent RNA Polymerase	C/T	Pro412Pro	synonymous	98.48%	7.7%
**17523**	ORF1ab	Helicase	G/T	Met429Ile	missense	98.50%	23.1%
**17690**	ORF1ab	Helicase	C/T	Ser485Leu	missense	98.02%	69.2%
**18877**	ORF1ab	3’-to-5’ exonuclease	C/T	Leu280Leu	synonymous	96.13%	30.8%
**22227**	S	Surface glycoprotein	C/T	Ala222Val	missense	99.09%	15.4%
**23403**	S	Surface glycoprotein	A/G	Asp614Gly	missense	61.39%	15.4%
**25563**	ORF3a	ORF3a protein	G/T	Gln57His	missense	71.27%	53.8%
**26559**	M	Membrane glycoprotein	C/T	Leu13Phe	missense	98.45%	15.4%
**28881**	N	Nucleocapsid phosphoprotein	G/A	Arg204Lys	missense	83.32%	30.8%
**28882**	N	Nucleocapsid phosphoprotein	G/A	Arg204Lys	missense	83.37%	30.8%
**28883**	N	Nucleocapsid phosphoprotein	G/C	Gly205Arg	missense	83.38%	30.8%

#### Structural analysis of SARS-CoV-2 spike glycoprotein and its mutations

A preliminary structural analysis of the spike glycoprotein mutations was performed. All the spike mutations were found to be in medium proximity to the human ACE2 receptor binding motif (RBM), with the exception of Ala222Val. Latter residues were located in closer proximity to the receptor-binding site. ConSurf analysis was used to decipher the evolutionary conserved and non-conserved regions based on the known spike sequences. As a statistically robust analysis method, ConSurf results demonstrated that all the described spike mutations occurred in moderate to highly variable regions except for the Asp614Gly mutation. The mutation Ala222Val was located in a region with the highest variability score.

### Molecular Dynamics (MD) simulations

**Fig 6:**
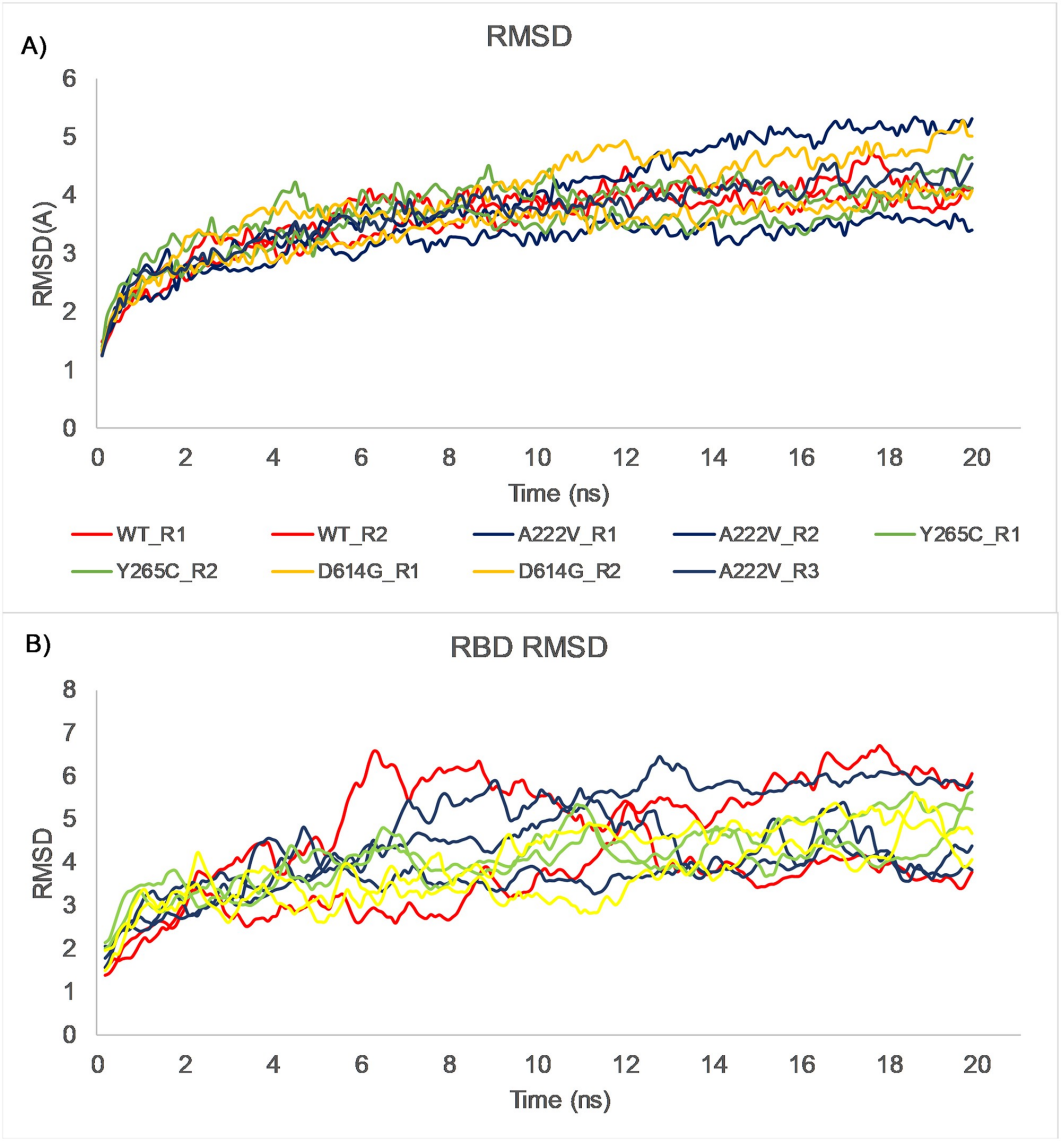
Molecular dynamics simulations. (A) RMSD of alpha carbon atoms of the reference SARS-CoV-2 spike protein (WT) and its single mutations (A222V, Y265C, and D614G) versus time at 310.15°K. (B) RMSD of alpha carbon atoms of the reference SARS-CoV-2 spike protein (WT) and its single mutations (A222V, Y265C, D614G) versus time at 310.15 K around the ACE-2 Receptor Binding Domain residues. Running averages of each RMSD value are plotted.

**Fig 7:**
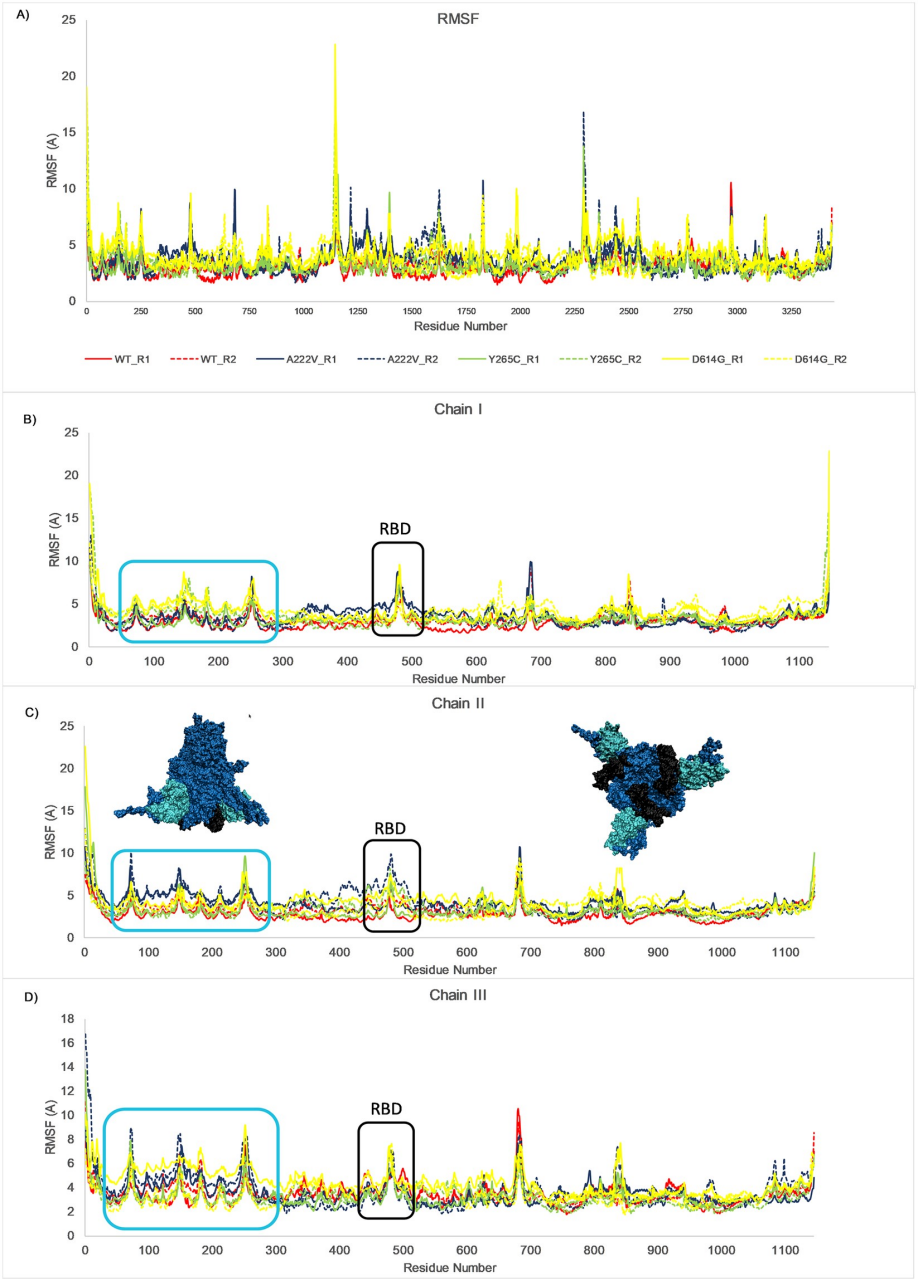
RMSF analysis over 20 ns trajectory. Per residue RMSF values averaged over 20 ns trajectory for (A) spike trimeric protein and its individual (B) Chain I, (C) Chain II. Reference trimeric spike protein 3D-surface rendering from side and bottom, showing the boxed regions, are shown in the inset with the same colors (snapshots exhibit the conformation at T = 20 ns), (D) Chain III. The black box indicates the Receptor Binding Domain residues. The cyan box indicates a highly fluctuating region in all three chains.

**Fig 8:**
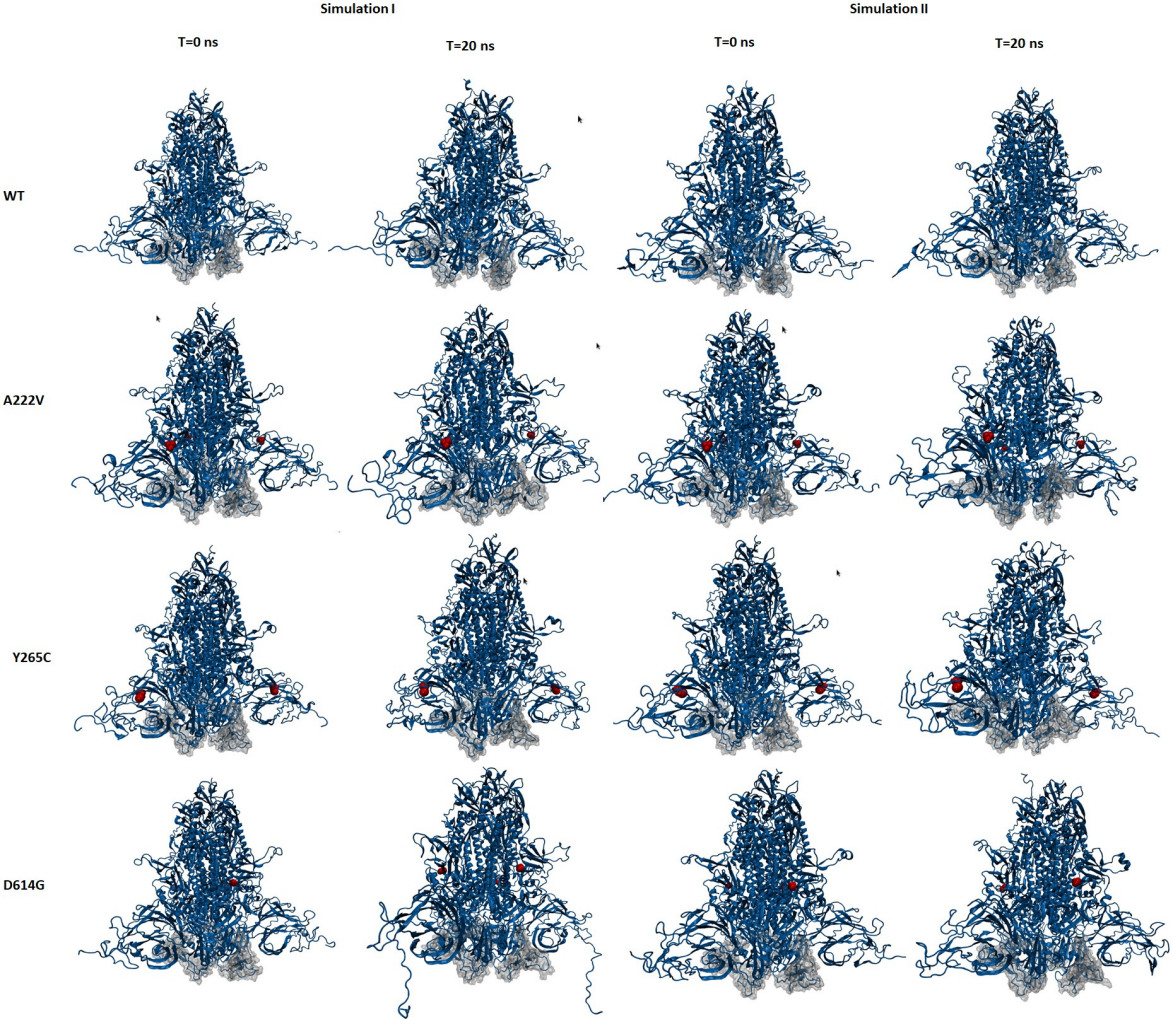
Side view of the trimeric spike protein at the start and end of the 20 ns MD simulation. Duplicate simulation results are shown. (A) WT, reference trimeric spike protein, (B) A222V mutant, (C) Y265C mutant, and (D) D614G mutant. Blue color: overall 3D structure, Silver color: RBD, and Red color: Residues mutated.

All-atom Molecular Dynamics simulations of the SARS-CoV-2 trimeric spike protein and single mutations were performed to understand the impact of these point mutations on the protein structure. RMSD analysis of the alpha carbon atoms over a 20 ns trajectory at 37°C is shown in Fig 6A. A RMSD comparison of the ACE-2 receptor-binding domain (RBD) residues is indicated in Fig 6B. At 37°C, a comparison of RMSD values for the reference structure (WT) and single mutations did significantly change. However, as shown in Fig 6B, RMSD values were also increasing for RBD residues throughout the simulations. This finding was also supported by RMSF analysis (Fig 7), showing the per residue fluctuations of the whole proteins and RBD regions at the end of the 20 ns simulation. The overall structure of the trimeric spike reference protein (aka WT) and the respective mutations, relative positions of the RBD regions at the start (t = 0 ns) and end (t = 20 ns) of the simulation are shown in Fig 8.

The solvent-accessible surface area, also known as SASA, with an average of at least two simulations for all structures over the 20 ns trajectory, is shown in S2A and S2B Fig, and S2 Table. All proteins showed a time-dependent increase in solvent accessible surface area during the 20 ns simulation period. However, the increase in the SASA of the Asp614Gly mutation was to a lesser extent. Moreover, a SASA calculation for the residues between 439 to 501 (including the RBD region) indicates that SASA change was minimal for the Asp614Gly mutation, which implies a more compact, structureless surface exposed with respect to the other mutations and reference protein regarding this region. Indeed, this compactness could be further observed in S2C and S2D Fig.

**Fig 9:**
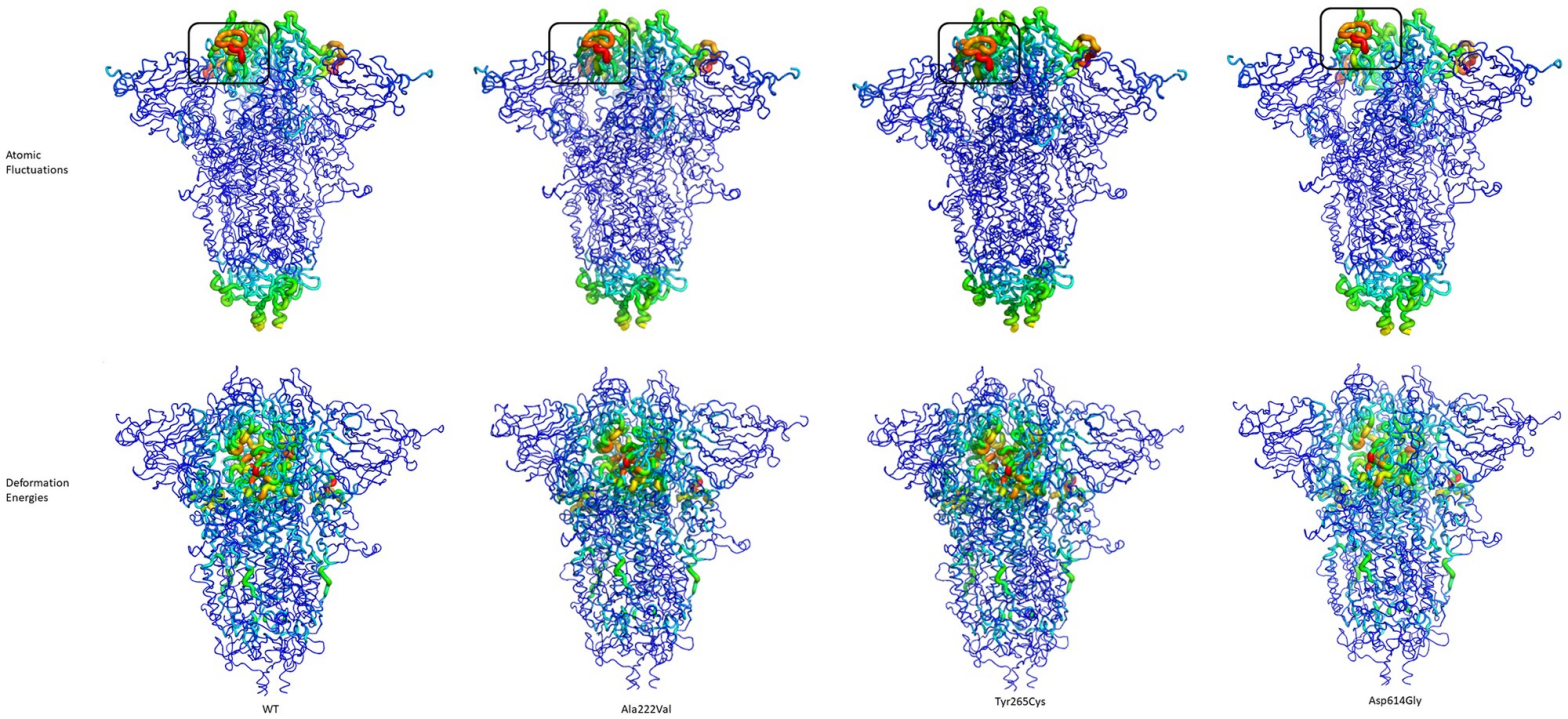
Enhanced normal mode analysis of the trimeric spike protein along the 20 ns MD simulation. WT, reference trimeric spike protein, A222V mutant, Y265C mutant, and D614G mutant. The top cyan row shows the atomic fluctuations, bottom row indicates deformation energies. Black box: residues 474–488 from RBD. The red color indicates higher fluctuations.

Enhanced Normal Mode Analysis of BIO3D was performed to compare and predict large motions in the spike protein upon mutation. It has been long known that most of the structural differences between two conformational states of a protein could be explained using a few normal modes with lower energy. In NMA, atomic fluctuations obtained from these normal modes were shown to match the experimental B-factors and used to predict the overall protein flexibility. For each mutant, the atomic fluctuations of the Cα atoms obtained from eNMA analysis and deformation energies were mapped to the spike 3D structure as shown in Fig 9. Atomic fluctuations mapped onto the 3D spike structure indicated a movement between residues 474 and 488 (Fig 9. boxed part) for all mutants. This region is located in the RBD domain and involved in the interactions with the ACE-2 receptor. Deformation analysis reflecting the local flexibility such as atomic motion with respect to the neighboring atoms exhibited similar local flexibility in the region just below the RBD structure. Although the fluctuation intensities and the location of the fluctuations were similar, residues 474–488 of the Asp614Gly mutant were mapped to a relatively more upward extended conformation [46, 48].

## Discussion

In Turkey, PCR testing of SARS-CoV-2 started soon after the pandemic began, whereas the viral genome sequence data started increasing by the end of 2020, recognizing “variants of concern” across the world. Hence, the number of submitted sequences between June-November 2020 is not high enough to reflect the actual distribution of different strains in Turkey. On the other hand, with the acceleration in viral genome sequencing by the end of November 2020, we were able to analyze the strain changes in Turkey. Here, in addition to the 13 cases from our clinic, the SARS-CoV-2 genomes of all the samples submitted through the end of May 2021 from Turkey to the GISAID database were analyzed.

With the high level of variability in the SARS-CoV-2 genome, strain classification became increasingly important. There are different classification strategies, developed by different organizations like PANGO lineage [23], GISAID [49], Nextstrain [50], Public Health England [51], and World Health Organization [20]. Although similar, each organization has its own algorithm and nomenclature in classifying the different viral strains. In this study, the data were analyzed according to the GISAID clades and PANGO lineages. There were seven clades and the only clade that was not detected was the V clade, which was shown to appear at the beginning and lost at later phases of the pandemic [52]. For the lineage analysis, the “variants of concern” that are detectable in our data were selected, namely, B.1.1.7 (Alpha), B.1.351 (Beta), P.1 (Gamma), B.1.617.2 (Delta), B.1, B.1.1. Additionally, we evaluated the B.1.9.5 lineage, which was detected at higher rates in Turkey than in other countries, and according to PANGO lineage, it was not found after May-June 2021 which was also in line with our finding that the B.1.9.5 lineage was not detectable in Turkey after May 2021. Between November 2020 and February 2021, all these lineages, except B.1.617.2, were detectable at high frequencies in Turkey, especially the B.1.1.7 strain, known as the UK variant. This may be partly due to the number of sequenced data and the number of detected cases, which were at the highest level in Turkey during the whole pandemic period. This period is known to be the second peak in our country and the average number of cases per day was 14110 [53]. During this time, the UK variant frequency showed a rapid increase and by the end of February 2021, the UK variant frequency showed a fast decrease. This decrease may be related to the start of the vaccination program in January 2021. The CoronaVac was the first vaccine to be applied in Turkey, with its effect later shown against the B.1.1.7 lineage [54]. Another important finding of our study was that all these lineage frequencies, except P.1, showed a decrease after February 2021. P.1 was in an increasing trend, and currently, it is the highest “variant of concern” in Turkey. P.1 was first detected in Brazil in December 2020 and it appeared in Turkish data by the end of November 2021. It was classified as a “variant of concern” by January 2021 and recently, it has been shown that the CoronaVac serum neutralization effect is lower against the P.1 lineage [54]. Since P.1 is still increasing in our country, lineage frequencies should be taken into consideration when designing vaccination programs.

The phylogenetic tree consists of data that is mostly obtained between December 2020-May 2020. Hence, the outer ring is mostly a projection of the last six months of the pandemic. Out of eight clades, four of them are derived from the G clade [55]. This is clearly detected in our phylogenetic tree as well. All the clades derived from G (GH, GR, GRY, and GV) were grouped as sub-branches of the G clade. GR was the most detected clade in our cohort, which was the most abundant clade in Europe, Asia, Africa, and South America. Our 13 genome sequences were also evaluated by the phylogenetic tree analysis and all the sequences, except coded ACUTG-5, fit in the O clade. g.GGG>AAC.28881_28883 group of single mutations, was the reason for discriminating the G clade from the GR clade, with genome sequence coded ACUTG-5 being the only case carrying this mutation.

In the analysis of 13 viral genome sequences from our clinic, among the 70 mutations, the cytosine to thymine change was the most common mutation type detected [56, 57]. In terms of gene region, most mutations were detected in the ORF1ab gene region (45%), which is in line with previously reported data [25, 26, 56]. The ORF1ab region encodes a helicase protein critical for viral replication and proliferation [58], but in another study from Turkey, it was found in only 8% of cases [25]. This discrepancy might be due to the regional differences between the two studies. Demir et al. samples were mostly derived from mid-Anatolia, whereas our patients reside in Istanbul.

The three consecutive single mutations (g.GGG>AAC.28881_28883) were always detected together, and in the literature, this change has been reported to affect transcription and replication of the virus by affecting the serine-arginine (SR)-rich motif in the nucleocapsid (N) protein [59]. On the other hand, the Asp614Gly missense mutation, which has been widely detected in different regions of the world [60], was seen in two (ACUTG-1 and ACUTG-5) out of the genome sequences of 13 patients in our cohort. This mutation is reported to cause the virus to be more virulent if seen on its own and/or in combination with other mutations [61]. A high infection rate was observed in the culture of the sample ACUTG-5, which is in line with previously reported data.

We also performed modeling of the mutations in spike glycoprotein and our analysis indicates that the trimeric spike glycoprotein surface becomes more hydrophilic upon the Ala222Val mutation. Although alanine to valine conversion conserves hydrophobicity to some extent, in general, alanine is known to be a better helix former and stabilizer in comparison to valine [62]. Further, molecular dynamics simulations of the trimeric spike protein and the mutations were performed to understand the mutations’ impact and verify the resultant effects. The positions of the mutations were found to be distant to the S1/S2 cleavage site, located between residues 680 to 687. It has already been shown that cleavage at S1/S2 is crucial for efficient viral entry into the target cells [63]. A Consurf analysis indicated that all the mutations were located in variable regions of the protein except for Asp614Gly. An RMSF analysis indicated two relatively large regions with the highest fluctuations, one located between residues 90 to 250 and the other region encompassed in the RBD. These regions were located at the bottom of the trimeric spike on the ACE-2 receptor interface.

The MD simulation results further corroborate an increase in the SASA of all mutations and the reference protein. The Asp614Gly mutant exhibited a lower SASA change than the reference protein and the other mutations, especially around the ACE-2 receptor-binding domain, resulting in a more compact region. A comparison of surface hydrophobicity of the trimeric spike reference protein with the Asp614Gly mutation over the simulation trajectory indicated surface hydrophobicity changed for both proteins. Thus, at the start of the simulations, both proteins’ surfaces were predominantly hydrophilic; by the midpoint of the simulation, the hydrophobic surface had increased due to the exposure of hydrophobic residues on the upper part of the structure and the RBD region. Part of the buried hydrophobic residues became exposed at the bottom of the protein, shifting it to a slightly open, aka “UP” conformation [64]. Over the last 10 ns of the simulation, both proteins shifted to a more closed, aka “DOWN” confirmation. All mutations exhibited a closed confirmation at the end. The MD analysis results showed a higher fluctuation in the ACE-2 receptor binding domain region in the reference protein and the single mutations. These results were further corroborated with eNMA analysis. Although these variations are not significantly different from the reference protein, one can speculate that these fluctuations might induce the SARS-CoV-2 spike proteins to shift to a slightly more open conformational state, aka “UP” form [64]. However, our conclusions are still limited in the sense that spike protein/mutations-ACE-2 receptor complex interactions have not yet been considered with these variations.

The most significant limitation in this study was the lack of information regarding the region and/or city, exact date, clinics, and patient outcomes. Also, the number of sequenced cases was not evenly distributed during the pandemic. There might be discrepancies between the sampling, transfer/storage, and or platform that were used in sequencing.

Currently, viral genome sequencing is ongoing worldwide and numerous genomes are being added to databases daily. These studies will help to discover new variations affecting protein structure, which might lead to differences in the virulence of SARS-CoV-2. Here, we evaluated the data of more than 4000 available SARS-CoV-2 genomes from Turkey regarding the clade and lineage classifications. Additionally, we analyzed our in-house data for variation types and their effects on protein levels. Compared to other countries, the amount of sequenced data from Turkey is not enough to make exact inferences. On the other hand, these findings provide an important output for the distributions of SARS-CoV-2 genomes around the country. Moreover, in light of these findings, new vaccination programs and/or prevention/treatment strategies against SARS-CoV-2 can be planned or developed.

## Supporting information

S1 Fig Bioinformatic workflow. Raw data were collected in a FASTQ format and their qualities were controlled with the FASTQC tool [65]. All FASTQ aligned to the NC_045512.2 sequence by Burrows-Wheels Alignment tool [66] SAM to BAM conversation was performed with Samtools [67]. PCR duplicates were removed with GATK [68] and variant calling was performed with GATK Haplotype Caller with ‘sample-ploidy’ option as 1. Mutations were confirmed with IGV.
(TIF)

S2 Fig Solvent Accessible Surface Area (SASA) values calculated at the start(T = 0) and end (T = 20 ns) of the MD simulation for (A) whole trimeric spike reference protein (WT) and mutants (A222V-Y265C-D614G), (B) RBD of the trimeric spike reference protein (WT) and mutants (A222V-Y265C-D614G), (C) Bottom view of the trimeric spike reference protein (WT) and D614G mutant at the start (T = 0 ns), midpoint (T = 10 ns), and end (T = 20 ns) of the 20 ns MD simulation. Average SASA values were calculated from duplicate simulation results. Blue color: overall 3D surface structure, Black color: the 3D surface of RBD, and Cyan color: the most fluctuating region in the structures.
(TIF)

S1 Table Metadata of the 4607 SARS-CoV-2 data which were uploaded to the GISAID from Turkey. Metadata includes collection dates of samples, lineage, GISAID clade, and amino acid substitutions information.
(XLSX)

S2 Table Solvent accessible surface area calculated for spike glycoprotein and respective mutations. (PDF)

S1 References (DOCX)
