# Supplementary figures and images for: Mutational landscape of SARS-CoV-2 genome in Turkey and impact of mutations on spike protein structure
Source: PLoS One. 2021 Dec 6;16(12):e0260438. doi: 10.1371/journal.pone.0260438 (PMC8648120; doi:10.1371/journal.pone.0260438)

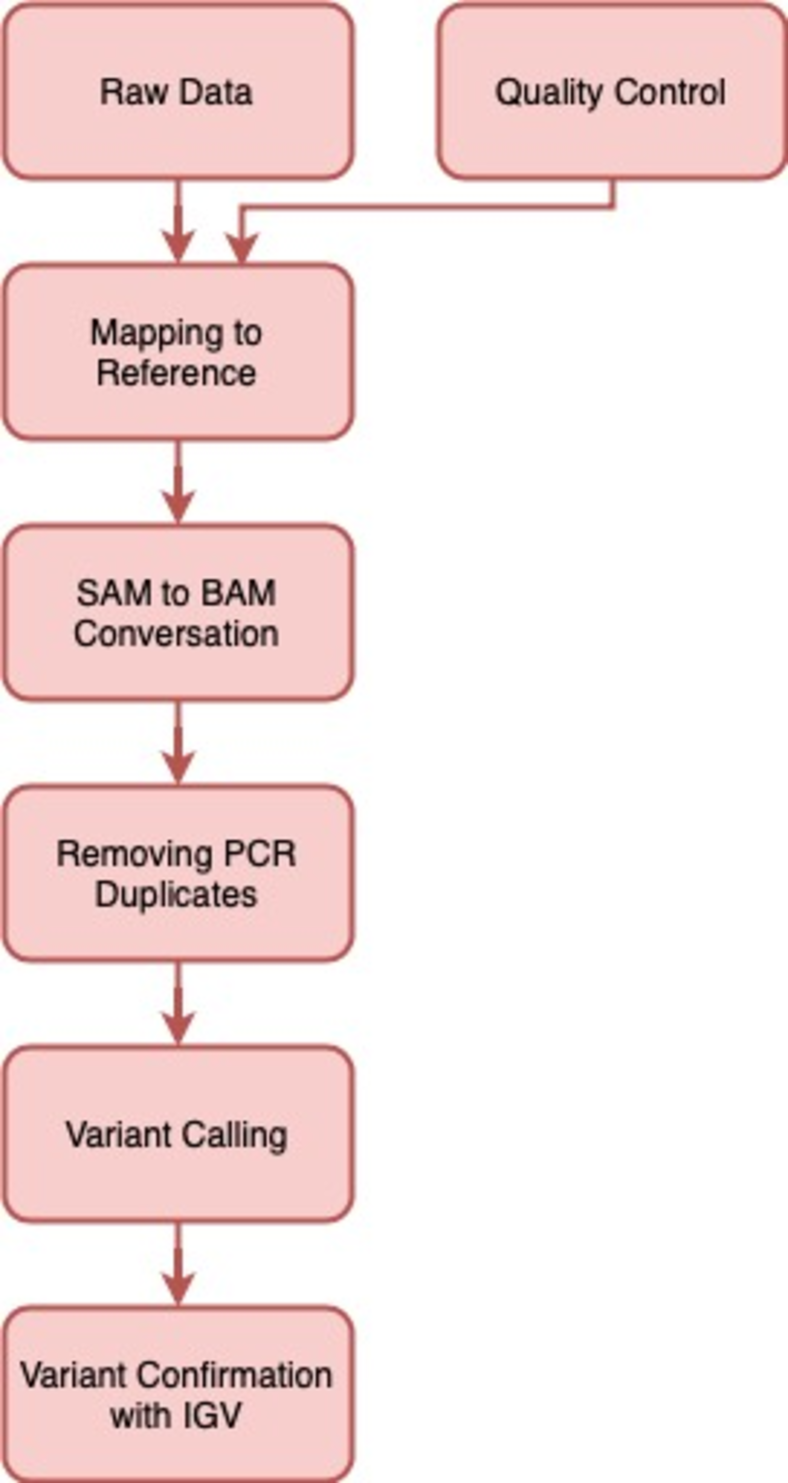

Supplement: S1 Fig — Raw data were collected in a FASTQ format and their qualities were controlled with the FASTQC tool [65]. All FASTQ aligned to the NC_045512.2 sequence by Burrows-Wheels Alignment tool [66] SAM to BAM conversation was performed with Samtools [67]. PCR duplicates were removed with GATK [68] and variant calling was performed with GATK Haplotype Caller with ‘sample-ploidy’ option as 1. Mutations were confirmed with IGV. (TIF) [file pone.0260438.s001.tif]

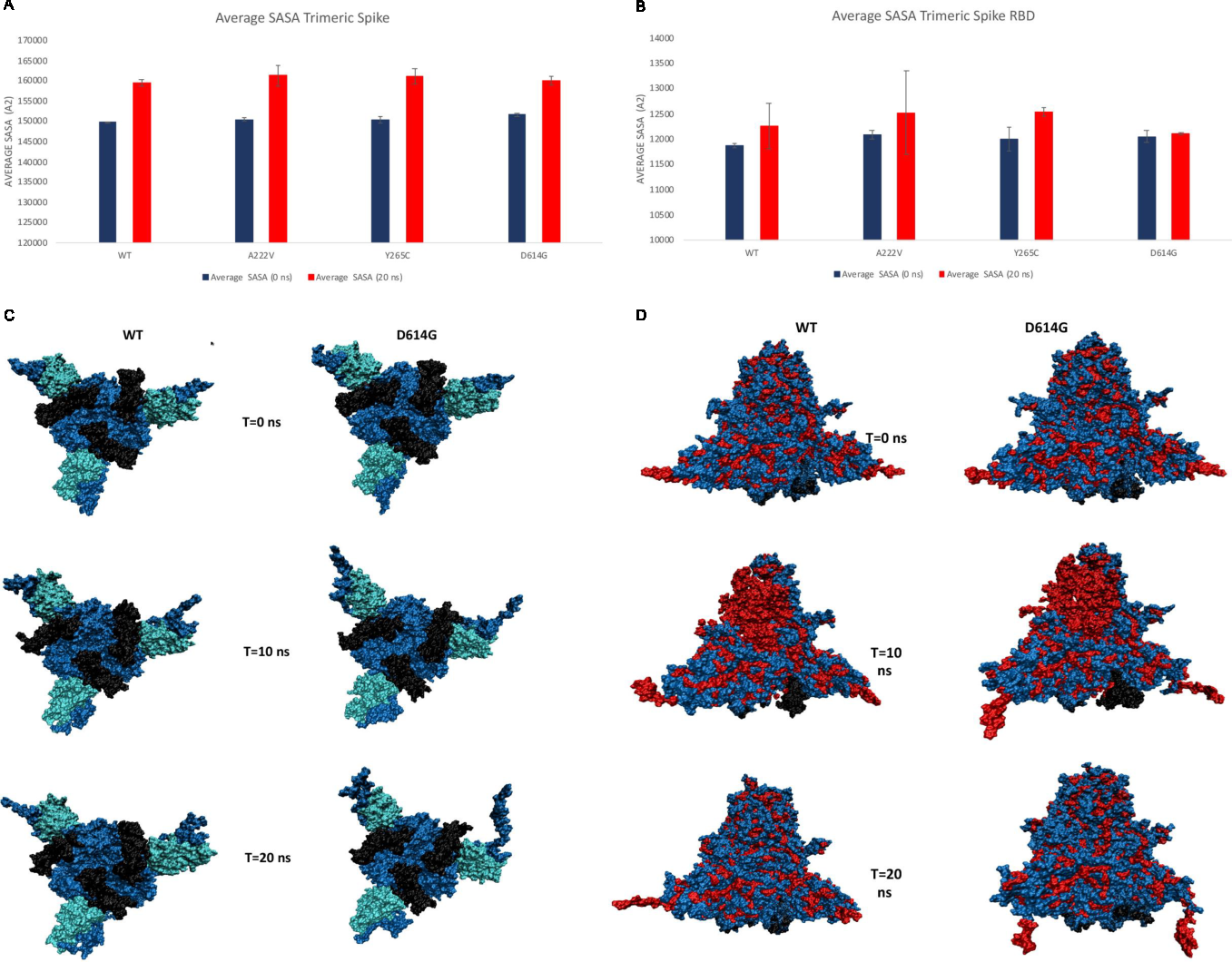

Supplement: S2 Fig — Solvent Accessible Surface Area (SASA) values calculated at the start(T = 0) and end (T = 20 ns) of the MD simulation for (A) whole trimeric spike reference protein (WT) and mutants (A222V-Y265C-D614G), (B) RBD of the trimeric spike reference protein (WT) and mutants (A222V-Y265C-D614G), (C) Bottom view of the trimeric spike reference protein (WT) and D614G mutant at the start (T = 0 ns), midpoint (T = 10 ns), and end (T = 20 ns) of the 20 ns MD simulation. Average SASA values were calculated from duplicate simulation results. Blue color: overall 3D surface structure, Black color: the 3D surface of RBD, and Cyan color: the most fluctuating region in the structures. (TIF) [file pone.0260438.s002.tif]
