# Supplementary material for: Mutational landscape of SARS-CoV-2 genome in Turkey and impact of mutations on spike protein structure
Source: PLoS One. 2021 Dec 6;16(12):e0260438. doi: 10.1371/journal.pone.0260438 (PMC8648120; doi:10.1371/journal.pone.0260438)
Supplement: S2 Table — (PDF) [file pone.0260438.s004.pdf]

**Variant**	6VXX	A222V	Y265C	D614G
**SASA (Å²)**	902286.9	902966.8	902575.9	902637.9
