# Supplementary material for: Mutational landscape of SARS-CoV-2 genome in Turkey and impact of mutations on spike protein structure
Source: PLoS One. 2021 Dec 6;16(12):e0260438. doi: 10.1371/journal.pone.0260438 (PMC8648120; doi:10.1371/journal.pone.0260438)
Supplement: S1 References — (DOCX) [file pone.0260438.s005.docx]

**Supporting information references**

65. Andrews S. FASTQC. A quality control tool for high throughput sequence data. 2010.

66. Li H, Durbin R. Fast and accurate short read alignment with Burrows-Wheeler transform. Bioinformatics. 2009;25(14):1754-60. Epub 2009/05/20. doi: 10.1093/bioinformatics/btp324. PubMed PMID: 19451168; PubMed Central PMCID: PMCPMC2705234.

67. Li H, Handsaker B, Wysoker A, Fennell T, Ruan J, Homer N, et al. The Sequence Alignment/Map format and SAMtools. Bioinformatics. 2009;25(16):2078-9. Epub 2009/06/10. doi: 10.1093/bioinformatics/btp352. PubMed PMID: 19505943; PubMed Central PMCID: PMCPMC2723002.

68. Poplin R, Ruano-Rubio V, DePristo MA, Fennell T, Carneiro MO, Van der Auwera GA, et al. Scaling accurate genetic variant discovery to tens of thousands of samples. 2017. doi: 10.1101/201178.
